# Changes in Antibiotic Resistance Gene Levels in Soil after Irrigation with Treated Wastewater: A Comparison between Heterogeneous Photocatalysis and Chlorination

**DOI:** 10.1021/acs.est.0c01565

**Published:** 2020-05-15

**Authors:** Ian Zammit, Roberto B. M. Marano, Vincenzo Vaiano, Eddie Cytryn, Luigi Rizzo

**Affiliations:** † Department of Civil Engineering, University of Salerno, Via Giovanni Paolo II 132, 84084 Fisciano, Italy; ‡ Department of Soil Chemistry, Plant Nutrition and Microbiology, Institute of Soil, Water and Environmental Sciences, Volcani Center, Agricultural Research Organization, Rishon LeZion 7505101, Israel; § Department of Agroecology and Plant Health, The Robert H. Smith Faculty of Agriculture, Food and Environment, The Hebrew University of Jerusalem, Rehovot 761001, Israel; ∥ Department of Industrial Engineering, University of Salerno, Via Giovanni Paolo II 132, 84084 Fisciano, Italy

## Abstract

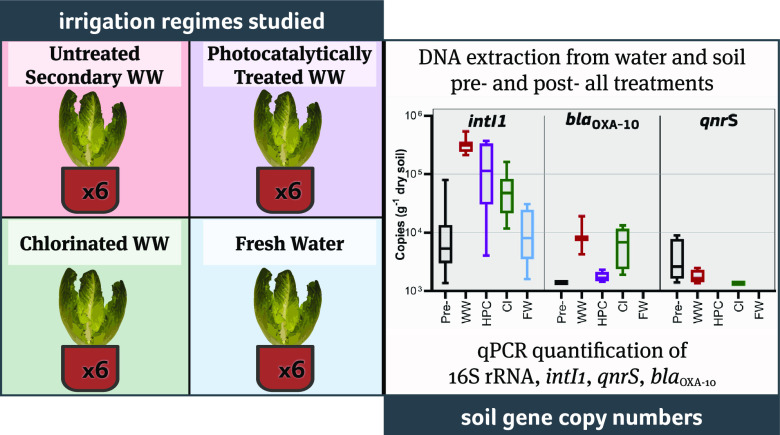

Wastewater (WW) reuse is expected to be increasingly indispensable in future water management to mitigate water scarcity. However, this increases the risk of antibiotic resistance (AR) dissemination via irrigation. Herein, a conventional (chlorination) and an advanced oxidation process (heterogeneous photocatalysis (HPC)) were used to disinfect urban WW to the same target of *Escherichia coli* <10 CFU/100 mL and used to irrigate lettuce plants (*Lactuca sativa*) set up in four groups, each receiving one of four water types, secondary WW (positive control), fresh water (negative control), chlorinated WW, and HPC WW. Four genes were monitored in water and soil, 16S rRNA as an indicator of total bacterial load, *intI1* as a gene commonly associated with anthropogenic activity and AR, and two AR genes *bla*_OXA-10_ and *qnrS*. Irrigation with secondary WW resulted in higher dry soil levels of *intI1* (from 1.4 × 10^4^ copies/g before irrigation to 3.3 × 10^5^ copies/g after). HPC-treated wastewater showed higher copy numbers of *intI1* in the irrigated soil than chlorination, but the opposite was true for *bla*_OXA-10_. The results indicate that the current treatment is insufficient to prevent dissemination of AR markers and that HPC does not offer a clear advantage over chlorination.

## 1 Introduction

Water scarcity is a growing global problem, and it is estimated that more than 3 billion people experience severe water scarcity for at least 3 months every year.^1^ The outlook is also not very reassuring; an increasing global population, climate change, and an increasing global standard of living and hence material consumption are set to further stress our water supplies.^2,3^ In arid regions, wastewater (WW) reuse is considered as an indispensable component in current water management strategies and possesses the scientific and political momentum to expand its current use to semiarid regions and developing countries.^4,5^ Treated wastewater (tWW) finds multiple uses, such as nonpotable urban uses, industrial water use, environmental and aquifer recharge, but most frequently, tWW is used in agricultural irrigation, especially in southern European countries, southwestern United States, Australia, and Israel, which have a strong agricultural sector and high water stress.^6^

The opportunity of expanding the scale of tWW reuse for agricultural irrigation comes with numerous potential issues. Some issues, such as soil salinity and hydrophobicity, are better understood.^7^ Other issues pertaining to wastewater reuse and the effects of organic pollutants (such as pharmaceuticals including antibiotics) and environmental antibiotic resistance (AR) dissemination are still in an early research phase.^8−10^ Antibiotic resistance is considered as one of the most urgent societal issues, which, if allowed to go unchecked, is forecast to become a major burden to the global economy and societal health and thus has been recognized as a priority issue by the United Nations.^11−13^ While the nosocomial dimension is expected to be the major hotspot of AR development and dissemination, the environmental dimension should not be ignored. Urban wastewater treatment plants (UWTPs) have been identified as environmental point sources for the dissemination of AR as they are linked, through discharge or reuse, to surface waters, groundwaters, and agricultural fields.^14^ UWTPs combine high bacterial loads in biological treatments and the presence of selective pressures—such as antibiotic compounds and heavy metals that can act as co-selectors.^15^ Routinely high levels of antibiotic-resistant bacteria (ARB) and antibiotic resistance genes (ARGs) are measured in UWTP effluents, making them point sources of environmental dissemination.^16−19^ While WW intended for reuse have higher quality requirements than the discharged effluents, UWTPs were not designed to mitigate AR and no regulation deals specifically with ARB or ARGs. Thus, a risk exists that the UWTP-resistome can find its way into the clinical resistome through the path of reclaimed wastewater.^20^ This could potentially take place both by horizontal gene transfer (from WW microbiota to soil microbiota) and through the establishment of resistant WW microbiota in the soil of edible crops. Expanding the frequency of wastewater reuse will inevitably increase the risk of this transfer. Regulations and guidelines for tWW reuse are often based on indicator bacterial loads. For example, a recent European Commission’s proposal^5^ for tWW intended for unrestricted crop irrigation set a maximum *Escherichia coli* load of 10 CFU/100 mL. This limit is also the same in Italian regulation for WW reuse.^21^

To meet this criterion, a disinfection step (tertiary treatment) is added, the most common and cost-effective of which is chlorination. Alternatives to chlorination are also well established since chlorination is known to form toxic byproducts, chiefly trihalomethanes, whose levels are also regulated.^22^ An additional drawback of chlorination, which is often not taken into account due to the lack of regulatory restrictions of AR indicators, is the fact that chlorination has been associated with an increase in the prevalence of antibiotic resistance.^23−25^ A possible alternative to chlorination or other consolidated disinfection methods are advanced oxidation processes (AOPs) such as heterogeneous photocatalysis (HPC). HPC is based on the formation of reactive oxygen species, and it has the potential to overcome the limitations of some conventional disinfection processes such as the formation of toxic disinfection byproducts (e.g., bromate and *N*-nitrosodimethylamine in ozonation (an AOP) and trihalomethanes in chlorination).^26,27^

The objective of our work is the comparison of different mechanisms of action on antibiotic resistance, one from a consolidated disinfection process (chlorination) through the action of HOCl and the other one from a nonconsolidated process, HPC (selected as model AOP) through the action of hydroxyl radicals. In this work, we compare, for the first time to our knowledge, changes of AR-associated genes in soil after irrigation with WW treated with HPC and chlorination, respectively, to evaluate the possible mitigation of AR transfer when these processes are used as a tertiary wastewater treatment for reuse in agricultural irrigation.

In particular, chlorination was applied through the addition of sodium hypochlorite and HPC using a previously optimized and trialed cerium-doped ZnO.^28,29^ Disinfection is carried out to reach the target of <10 CFU/100 mL of *E. coli*. Four irrigation regime groups composed of six lettuce plants (*Lactuca sativa* cultivar: Romaine), each set up and irrigated with one of four water types, namely, chlorinated tWW, HPC tWW, secondary WW (positive control), and fresh water (negative control). Water samples were taken before and after treatment, and soil samples before and after the irrigation campaign. DNA was extracted for the quantitative polymerase chain reaction (qPCR) analysis to quantify selected genes (*bla*_OXA-10_, *qnrS*, *intI1*, and 16S rRNA). *intI1* was chosen as it is an abundant tWW-associated gene that is linked to anthropogenic pollution and antibiotic resistance,^30,31^*qnrS*, a plasmid-associated ARG^32,33^ that confers moderate resistance to fluoroquinolone antibiotics known to be profuse in both human pathogens and wastewater, while *bla*_OXA-10_, a β-lactamase, was chosen on the basis of the fact that it is strongly associated with wastewater but not commonly found in soil.^34^ Hence, an increase in this gene following tWW irrigation indicates that it probably originated from tWW irrigation. Moreover, a common tWW-associated gene, such as is *intI1*, was included in the analysis for two additional reasons. First, to assess if <10 CFU/100 mL of *E. coli* alone is a suitable and informative indicator of water quality vis-à-vis AR dissemination during tWW reuse; second, if reaching this target (<10 CFU/100 mL of *E. coli*) through chlorination or HPC results in significant differences in *intI1* soil levels as an ARG-proxy gene representative of the tWW resistome. Due to the possible effects of disinfection byproducts (chlorination) and oxidation intermediates (HPC) on irrigated crops, plant aerial height and dry weight were also measured to evaluate phytotoxicity.

## 2 Materials and Methods

### 2.1 Lettuce Crop Setup

Sandy soil from Rehovot (Israel) which, prior to the study, was never irrigated with treated wastewater, was collected, sieved through a 1 mm mesh, and thoroughly homogenized. The physicochemical properties of the soil were previously characterized (see ref (35)). Twenty-four 3 L (15 cm base circumference) plastic pots were filled with approximately 3.3 kg of dry soil, and one lettuce (*L. sativa* cultivar: Romaine) seedling was transplanted into each pot. Pots were labeled by one of four series (water types to be irrigated with and a sequential number) and distributed randomly over the growing area inside a greenhouse at the Agricultural Research Organization in Rishon LeZion (Israel). Each one of the four groups was manually irrigated through a container (by pouring the volumes specified in Table S1 in the Supporting Information (SI)), these being secondary WW (positive control), fresh water (negative control), chlorinated WW, and photocatalytically treated WW. The plants were grown for a total of 55 days, starting in late October 2018 with daily temperature averages (day–night) for the entire growing period in the 16–25 °C range. Each pot received the same quantity of water and fertilizer as listed in Table S1 in the Supporting Information (SI). Plants were kept out of direct sunlight, and greenhouse air humidity was not controlled.

All plants were initially irrigated for 17 days (of the 55 days total) with fresh water (tap water without further treatment) to equilibrate autochthonous bacterial communities and reduce stress for the plants. Fresh water was tested for residual chlorine using MQuant active chlorine DPD kit (Merck Millipore) and was found to be lower than the detection limit of 0.1 mg/L. Each pot received the same quantity of water and, on selected days, nitrogen–phosphorus–potassium (NPK) fertilizer (at 55 mg/L total N), as listed in the irrigation log (Table S1).

### 2.2 Wastewater Sampling

Secondary treated wastewater was obtained from the Dan Region UWTP (Shafdan) in Rishon LeZion (Israel), which treats 400 000 m^3^/day of WW from the Greater Tel Aviv area (2.5 million population equivalent). The UWTP operates through an activated sludge process with hydraulic retention times in the aeration tank of ≈13 h and phosphorus removal via anaerobic and aerobic zones. WW to be used for the entire irrigation campaign (150 L) was sampled in two sessions, on the 2018-11-04 (WW1) and a second time on the 2018-11-25 (WW2); the parameters are presented in Table 1.

**Table 1:** Wastewater Characteristics of the Secondary Effluent as Sampled from the Shafdan UWTP^c^^,^^d^

parameter	WW1	WW2
chemical oxygen demand (COD)^b^	40 mg/L	34 mg/L
biological oxygen demand (BOD_5_)^b^	6 mg/L	7 mg/L
dissolved organic carbon (DOC)^a^	9.2 mg/L (unspiked)	8.9 mg/L (unspiked)
spiked = 10 μL of bacterial stock per liter of wastewater	11.9 mg/L (spiked)	10.7 mg/L (spiked)
dissolved total carbon^a^	51.0 mg/L (unspiked)	43.1 mg/L (unspiked)
spiked = 10 μL of bacterial stock per liter of wastewater	55.0 mg/L (spiked)	44.9 mg/L (spiked)
total nitrogen (TN)^a^	16.2 mg/L	14.4 mg/L
total suspended solids (TSSs)^a^	6.1 mg/L	7.0 mg/L
absorbance at 365 nm	0.0634 A	0.0698 A
1 cm path length^a^		
turbidity (NTU)^b^	2.2	2.7
pH^b^	7.4	7.5
unspiked *E. coli* load^a^	667 CFU/mL	467 CFU/mL
unspiked other coliforms load^a^	3300 CFU/mL	2567 CFU/mL

a Self-measured.

b Provided by Shafdan WWTP.

c Unspiked = WW measured as sampled.

d Spiked = WW after the addition of the bacteria stock of Section 2.3.

The sampled WW1 was stored in the dark at 4 °C, and weekly subsamples were taken for treatment and irrigation up to a maximum of 3 weeks. Stored at these conditions, the abundance of *E. coli* in the sampled WW was within half an order of magnitude throughout the 3 weeks. After these first 3 weeks, WW2 was collected and stored under the same conditions and used thereon for treatment and irrigation.

### 2.3 Preparation of a Bacterial Stock

From freshly sampled WW1, one part of WW was added to 19 parts of sterile Luria–Bertani (LB) broth in culture tubes and incubated overnight at 30 °C under constant shaking (180 rpm). The culture tubes were then centrifuged at 1000*g* for 5 min, the liquid was discarded, and the pellets were resuspended in 0.85% NaCl and combined to concentrate by a factor of 8 from the original LB broth concentration. The combined resuspended pellets were again centrifuged at 1000*g* for 5 min to remove any residual LB broth, resuspended in 50% glycerol/water, well homogenized by vortexing, and split and stored in separate vials at −80 °C for weekly spiking of wastewater prior to starting treatment.

### 2.4 Bacterial Enumeration

Bacteria were enumerated on Chromocult Coliform Agar (Merck Millipore) after appropriate dilution in 0.85% NaCl and filtration on 0.45 μm cellulose nitrate membranes (Sartorius Stedim). *E. coli* and other coliforms are differentiated on the selective agar by the color of the colonies (according to ISO 9308-1:2014). For bacterial enumeration post treatment, where the goal was to achieve <10 CFU/100 mL of *E. coli*, 100 mL of undiluted WW was filtered and plated. Positive controls were performed, and all measurements were carried out in triplicate.

### 2.5 Synthesis of Photocatalyst

Cerium-doped zinc oxide was prepared and characterized according to previous published methods.^28^ In brief, cerium-doped zinc oxide at 0.04:1 Ce/Zn was synthesized via the hydroxide-induced hydrolysis of zinc nitrate in the presence of Ce(III). X-ray diffraction (XRD) was measured using an X-ray micro diffractometer Rigaku Dmax-RAPID, using Cu Kα radiation (spectrum provided in Figure S1, SI), and Raman spectroscopy was measured at room temperature with a Dispersive Micro Raman (InVia, Renishaw) equipped with a 514 nm laser in the range of 200–2000 cm^–^ Raman shift.

### 2.6 Disinfection Procedure

Disinfection was carried out weekly. As dictated by the weekly required analyses and hence water volume, 6.5–7.5 L of WW was subsampled from the stock stored at 4 °C and brought to room temperature. To approximately double the bacterial load from the autochthonous level, 10 μL of bacterial stock (prepared in point 2.3) per liter of WW was spiked and well mixed inside a rectangular poly(ethylene terephthalate) (PET) tank of 54 cm × 21 cm. Bacterial enumeration before and after spiking was carried out with every single disinfection process. For photocatalytic disinfection, 0.1 g of Ce–ZnO per liter of WW was weighed and suspended in a minimal volume of sterile water and sonicated, for 5 min, using a QSonica Q125 (CT) probe sonicator at an amplitude of 70% of the maximum. The photocatalyst was then added to the WW and allowed to equilibrate for 30 min in the dark under constant stirring to keep the powdered catalyst suspended. Five minutes before this dark period was over, two Osram Dulux L BL UVA 55W/78 lamps coupled to an Osram Quicktronic Professional Optimal ballast were warmed up and subsequently placed at a distance of 35 cm from the bottom of the rectangular tank. The photocatalytic process was kept for a total of 3 h, after which bacterial enumeration post treatment was carried out and tWW was decanted leaving the powdered photocatalyst on the bottom. A portion of this tWW was used the same day for irrigation while the rest was stored at 4 °C to be used in the 4 days that followed disinfection.

Similarly, WW was treated with chlorination weekly, 6% sodium hypochlorite was diluted 10-fold, and its concentration was verified using MQuant active chlorine test strips (Merck Millipore). A suitable quantity to achieve an initial concentration of 2 mg/L of active chlorine was added to 6.5–7.5 L of WW under constant stirring as required for that week. The water was sampled 5 min after adding hypochlorite and after 90 min. The concentration of active chlorine added to the WW was tested with MQuant active chlorine DPD kit (Merck Millipore). The initial measured concentration was in the range of 1.8–2 mg/L, while the concentration after 90 min was always <0.2 mg/L; residual active chlorine was not quenched as such low levels are allowed by Italian regulation and is even lower than WHO drinking water recommendations.^36,37^ As was the case for HPC-treated WW, a portion was used the same day for irrigation while the rest was stored at 4 °C to be used in the 4 days that followed disinfection.

### 2.7 Water Samples—Preparation and DNA Extraction

Water samples were filtered through a 0.45 μm membrane (Sartorius, Göttingen, Germany) to be processed for DNA extraction and subsequent qPCR analysis. Water samples were taken (i) directly after sampling from the UWTP, (ii) before disinfection but after spiking (10 μL per 1 L of WW) with the bacterial stock of Section 2.3, and (iii) after both disinfection methods. A volume of 250 mL was filtered for the secondary WW samples, while 300 mL was filtered for the tWW samples. Additionally, 500 mL of fresh water that was supplied to the negative control group was also sampled and analyzed.

The membranes used for filtering each sample were stored at −80 °C until processed for DNA extraction using DNeasy PowerWater Kit (Qiagen, Hilden, Germany). The provided instructions were followed without modifications: the final elution volume was 100 μL, which was divided into aliquots and stored at −80 °C.

### 2.8 Soil Sample—Preparation and DNA Extraction

Soil was sampled before commencing the irrigation campaign and at the end of it because the accumulation of integron genes and ARGs in the soil is expected to be higher at the end of the irrigation period. Preirrigation sampling was taken after all pots were irrigated for 17 days with fresh water (point 2.1), while post-tWW irrigation sampling was carried out 55 days after transplanting and 24 h after the last irrigation took place. A total of 48 soil samples were taken from the top layer up to a depth of 3–5 cm of soil inside the pot, taking into account that: (i) together with the microbial communities of the rhizosphere, the topsoil is the most metabolically active portion of soil and the part expected to be more effected by the water type; (ii) topsoil is also where the targeted tWW-borne genes (and their related bacterial hosts) would most likely be present. For lettuce, it is also the only part that can be in contact with the edible part of the plant (e.g., wind, or splatter during irrigation) and could be contaminated by topsoil. Sampling was carried out by thoroughly mixing the soil and putting >15 g of soil into a sterile 50 mL Falcon tube.

DNA extraction was carried out using 250 mg of soil and processing with Qiagen’s DNeasy PowerSoil Kit (Hilden, Germany). For the initial lysis step, an MP Biomedicals FastPrep-24 Classic (CA) homogenizer was used; two cycles at a speed of 5 m/s for 23 s with a gap of 5 min between homogenizing cycles to avoid overheating. The final elution volume was 100 μL, which was split and stored at −80 °C.

### 2.9 Quantitative Real-Time PCR Analysis

The gene copy number was quantified according to previously employed methods.^38^ In summary, a total of four genes were analyzed by qPCR, 16S rRNA, *intI1*, *qnrS*, and *bla*_OXA-10_. Two plasmids were used as templates for standard curve calibration, pMARPAT for *bla*_OXA-10_(38) and pNORM1^39^ for all of the other genes. The plasmids were extracted from fresh bacterial cultures using QIAprep Spin Miniprep Kit (Qiagen, Hilden, Germany) and enzymatically linearized with EcoRI (Thermo Scientific, MA) prior to use. Plasmid extracts were quantified using a Qubit 2.0 fluorometer (Thermo Fisher Scientific, MA) and the dsDNA BR assay kit (Thermo Fisher Scientific, MA).

All of the herein reported procedures for qPCR analyses were conducted in accordance with Bustin et al.^40^ Copy number quantifications were carried out in duplicate together with a negative control (i.e., no DNA template PCR-grade water) on a 96-well plate using a StepOnePlus real-time PCR running StepOne software v2.3 (Applied Biosystems, CA). FAST SYBR Green MasterMix (Thermo Scientific, MA) was used to amplify the 16S rRNA, whereas POWER SYBR Green MasterMix (Thermo Scientific, MA) was used for *bla*_OXA-10_, *qnrS*, and *intI1* genes. Each well contained 10 μL of the respective Mastermix, 1 μL of sample extract, and 0.5 μM of both the reverse and forward primer, making up a total well volume of 20 μL. Other program parameters are according to Marano et al.^38^ and Supporting Information Table 4 therein. In each run, an inhibitor test was included for each sample type (soil, and each of the four types of waters), as suggested by Bustin et al.^40^ by means of an additional 10-fold dilution.

Reported results had an efficiency of 100 ± 10% and *R*^2^ values greater than 0.99. Results for water samples are expressed as copy numbers per volume of filtered water, while those from soil samples are expressed as copy number per gram of dry soil. The limit of quantification (LOQ) values in soil and water samples were defined considering the minimum copy number quantifiable by the qPCR procedure (three copies according to Bustin et al.^40^), elution volumes in DNA extraction, sample volume/mass, and other parameters such as dilution, eventually accounting for 1200 copies/g of dry soil and 0.6 copies/mL of water.

### 2.10 Auxiliary Methods

Dissolved organic carbon and total nitrogen were measured on a Shimadzu TOC-V analyzer (Kyoto, Japan). Total suspended solids were measured by filtering 300 mL of WW and weighing mass differences after drying at 105 °C, accounting for mass changes in a blank membrane. Soil dry mass was measured according to ASTM D 2216-10 but modified to use 5 g of soil; the weight was stable after 24–36 h at this temperature (110 °C). Plant aerial height was measured as the part of the plant from the soil to the topmost part extended perpendicularly upward. Plant dry weight was measured by cutting the entire aerial height and drying the plants individually at 80 °C for 36–48 h. One-way analysis of variance (ANOVA) (α = 0.05, *n* = 6 per water type) was performed in GraphPad Prism 8 (CA) on the two metrics separately to test for significance.

## 3 Results

On a weekly basis and prior to every disinfection procedure, *E. coli* and other coliforms were enumerated in the freshly spiked wastewater (Table 2).

**Table 2:** WW Bacterial Densities prior to Disinfection Tests

	mean	SD	max	min	*n*
*E. coli* (CFU/mL)	1529	954	3600	350	31
other coliforms (CFU/mL)	3777	1317	6550	2200	31

This water was used for irrigation as is for the spiked wastewater series as well as used as feed WW for disinfection with both HPC and chlorination to the target of <10 CFU/100 mL of *E. coli*. Bacterial regrowth of treated wastewater was not an issue when stored at 4 °C. The *E. coli* loads of this stored tWW never exceeded the established limit (<10 CFU/100 mL of *E. coli*) even after 5 days of storage (i.e., the maximum storage time before a fresh batch was treated for the following week of irrigation).

**Figure 1:**
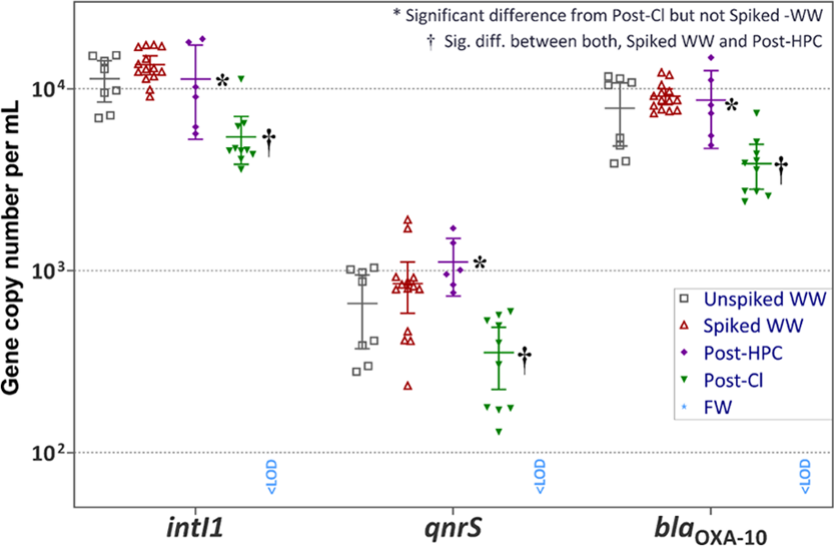
Gene copy numbers of water samples per milliliter of treated water. For chlorination (post-Cl), all three genes were statistically significantly lower than the starting wastewater (spiked WW) and post-HPC, while post-HPC was only different from post-Cl. Error bars = 95% confidence interval (C.I.).

As for qPCR results, Figure 1 shows the abundance of the three monitored genes in water samples, including before and after spiking with additional bacteria and before and after both disinfection treatments. Table 3 shows the measured 16S rRNA gene copies normalized by water volume. These were quite similar among all WW samples, both before and after treatment. Fresh water samples had 3 orders of magnitude lower 16S rRNA copy numbers than WW samples. Statistical differences as tested with a one-way ANOVA are shown within Table 3.

**Table 3:** Bacterial Abundance in Water Samples Based on qPCR-Derived 16S rRNA Gene Copy Numbers

	16S rRNA gene copies per milliliter of water in				
	(A) unspiked WW	(B) spiked WW	(C) chlorination	(D) HPC	(E) fresh water
mean	1.44 × 10^6^	2.02 × 10^6^	1.17 × 10^6^	1.74 × 10^6^	2.05 × 10^3^
SD	6.33 × 10^5^	4.88 × 10^5^	3.40 × 10^5^	4.43 × 10^5^	1.84 × 10^3^
significant difference with group(s) (*p* ≤ 0.05)	B, E	A, C, E	B, E	E	A, B, C, D

As for *intI1*, *qnrS*, and *bla*_OXA-10_, these genes were all detected in secondary WW samples at levels very similar to what Marano et al.^38^ reported. As was the case for *E. coli* and other coliform densities, spiking only increased the prespike values negligibly if at all, since it was not statistically significant. However, this was carried out to achieve baseline bacterial abundances and gene copy numbers among the different weeks of use and the two different WW samples, rather than to increase them substantially. As was the case with 16S rRNA, the HPC treatment did not significantly impact the abundance of any of the three antibiotic resistance-associated genes under the given conditions, while chlorination did result in a statistically significant albeit small decrease in gene copy numbers per unit volume (Figure 1) of all three genes (*intI1*, *qnrS*, and *bla*_OXA-10_) in the water phase.

**Figure 2:**
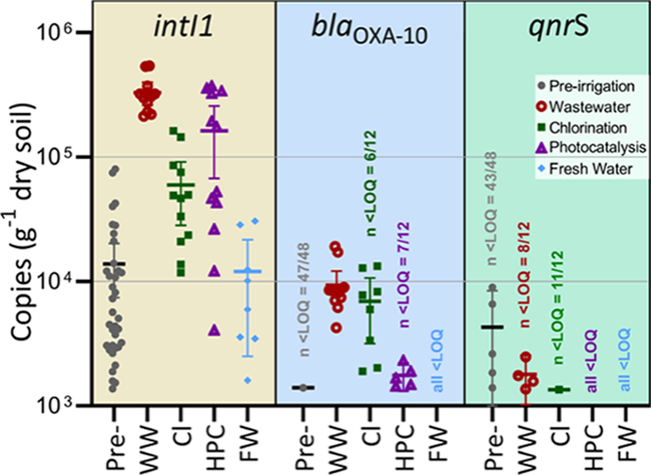
Gene copy numbers of soil samples per gram of dry soil. Preirrigation samples (in gray) represent the soil before they started receiving their respective water type in each of the four groups (WW, Cl, HPC, FW). These four groups all show the soil levels after 38 days of irrigation. For *IntI1*, both Cl and HPC are statistically significantly lower than wastewater irrigation, while for *bla*_OXA-10_, only HPC was lower. For both genes, soil levels after irrigations were statistically significantly higher than preirrigation levels. Error bars = 95% C.I.

Each of the 48 soil samples was analyzed for the same genes to assess the effect WW irrigation has on their presence and potential accumulation in the fresh water and treated WW soils. The abundance of 16S rRNA per gram of dry soil increased only between pre- and post-irrigation levels for the secondary WW-irrigated series (*t*-test *p* = 0.0044; 144% increase in post irrigation), while no significant changes in 16S rRNA levels at the end of the irrigation campaign were measured for chlorination (*p* = 0.5022), HPC (*p* = 0.6752), and fresh water (*p* = 0.3037) irrigation. Figure 2 shows the qPCR results from soil samples as gene copies per gram of dry soil of *intI1*, *bla*_OXA10_, and *qnrS*. Preirrigation samples (Figure 2, pre-) show the copy numbers per gram of dry soil of the 24 pots before splitting into four groups and irrigating with one of four water types (i.e., WW—wastewater, Cl—chlorinated, HPC—photocatalysis, and FW—fresh water). Irrigation with WW was carried out as a positive control, i.e., to link the presence of the studied genes in the water used for irrigation to the levels in soil. As shown in Figure 2 (WW), this was in fact the case for *intI1* (*p* ≤ 0.0001), while the WW post-irrigation levels for *bla*_OXA10_ were also higher than the preirrigation quantities, the latter of which were all but 1 below the quantification limit. On the other hand, no evidence for an increase in soil copy numbers was found for *qnrS*, which was present in water at 1 order of magnitude lower levels than the two other genes (Figure 1).

The fresh water-irrigated soil series resulted in the lowest measured levels of all genes, and mostly below quantification levels. This soil series received only fresh water, and these genes were not found in this water type at levels above the quantification limit (Figure 1). Both treatments showed higher statistically significant values (in each case in terms of copies per gram of dry soil) compared to preirrigation levels for *intI1* (chlorination and HPC both *t*-test *p* ≤ 0.0001). While *bla*_OXA10_ was below LOQ at the preirrigation stage in most samples, it was detected at low levels in a number of samples both in the chlorinated and HPC series (Figure 2). No evidence for enrichment was observed for *qnrS* when irrigating with HPC- or Cl-treated WW compared to the preirrigation values.

Both treatments did show statistically significantly lower levels of *intI1* relative to WW irrigation (Cl *p* ≤ 0.0001; HPC *p* = 0.0048). Such effect of both treatment methods also seems to take place for *bla*_OXA10_, since postirrigation levels are more frequently below LOQ for the two treatments.

Looking only at the quantity of genes received by each pot throughout the irrigation campaign, one can infer an indication of the copy numbers of genes needed during irrigation to cause increases in soil copy numbers of the same genes. Over an irrigation period of 37 days, each pot containing 3.3 kg of dry soil received a total of 3730 mL of WW.

Not surprisingly, no increase was observed in soil for the gene supplied in smallest quantities in water, i.e., *qnrS*. While the quantity of water supplied for irrigation was often close to the holding capacity (≈242 mL/kg of dry soil) of the soil, any stratification in the bacteria and ARGs in the soil would not be taken into account by the average values reported in Table 4 since sampling was carried out on the top 5 cm of soil. Thus, it should be considered as more as a minimum possible value rather than an average at which ARG increases are observed.

**Table 4:** Quantity of the Respective Genes Received through Water Per Gram of Dry Soil throughout the Entire Irrigation Campaign

	*intI1*	*qnrS*	*bla*_OXA-10_
average copy number in WW per milliliter	1.36 × 10^4^	8.49 × 10^2^	9.10 × 10^3^
total copy number received over 37 days	5.06 × 10^7^	3.17 × 10^6^	3.39 × 10^7^
total copy number per gram of soil	1.53 × 10^4^	9.60 × 10^2^	1.03 × 10^4^

**Figure 3:**
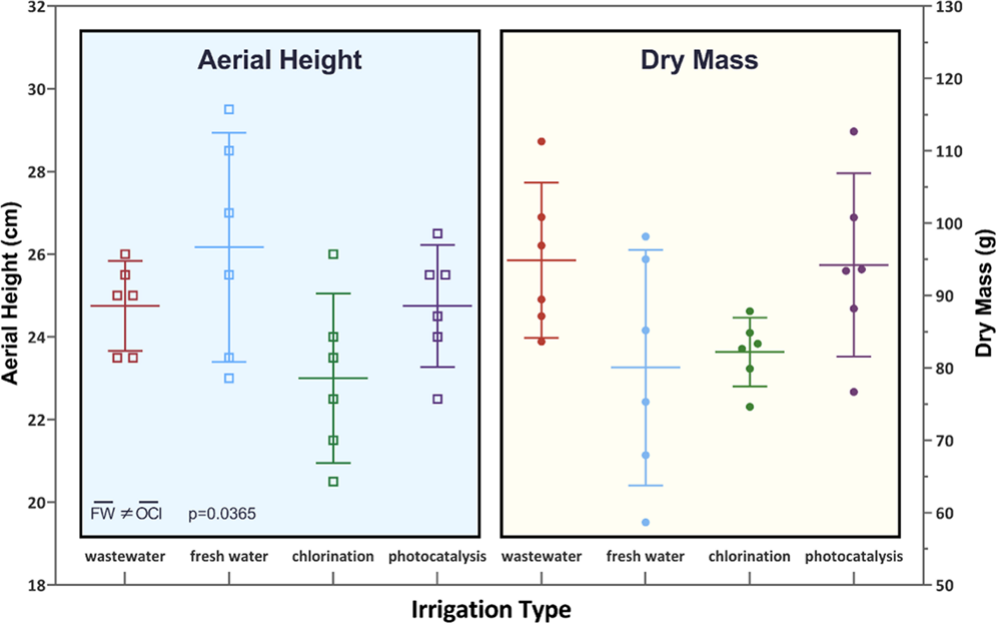
Plant growth metrics. Error bars = 95% C.I.

Plant growth was also monitored through aerial height and dry mass measurements (Figure 3), to assess phytotoxicity and other detrimental effects on crop growth with the different water regimes. The only statistically significant (*p* = 0.0365) difference in either plant growth metric was recorded between the average value of fresh water-irrigated plants (26.2 cm) and chlorinated wastewater plants (23.0 cm). However, the dry masses of the plants in these two groups were not different (*p* > 0.05) (Figure 3).

## 4 Discussion

While it is commonly reported in the literature that the degradation of selected bacterial genes in wastewater using various disinfection processes under real or realistic conditions is low,^31,41−43^ this was especially the case herein. The intensities of treatments used were kept at realistic levels, and this may explain the observed persistence of genes in the treated water samples. AOPs such as photocatalysis and ozonation have been shown to be able to reduce gene loads by a few orders of magnitude depending on the intensity of treatment.^44,45^ Ozonation is the most promising treatment when considering only the degradation of genes. Iakovides et al.^45^ used 0.75 g O_3_/g DOC and a retention time of 40 min to show a reduction by 4 orders of magnitude of 16S rRNA and up to 5 orders of magnitude of *IntI1* for both genes in terms of gene copy number per unit volume. Lower intensities are less effective, in the same study, lowering the dose to 0.25 g O_3_/g DOC and retention time to 10 min, resulted in 2 orders of magnitude lower removal of 16S rRNA and 3 orders of magnitude lower removal of *IntI1* (in both cases in terms of gene copy number per unit volume of treated water) compared to the aforementioned higher dose. Photocatalysis employed in a continuous system with 20 W of UVA (albeit using higher-efficiency light-emitting diode (LED) than the compact fluorescent tubes used herein) with a retention time of 26 min and a water volume of 0.23 L gave a reduction in 16S rRNA (3 orders of magnitude per unit volume) and *IntI1* (4 orders of magnitude per unit volume).^44^ UV-C disinfection treatments at real scale in UWTPs have shown poor removal of ARGs.^41,46^ Chen and Zhang^46^ studied six ARGs together with *IntI1* and 16S rRNA in three UWTPs in China operating different processes. They showed that UWTP operating UV-C disinfection had lower log removals than constructed wetlands and even biological aerated filters. UV-C disinfection is also very dependent on the target gene. McKinney and Pruden^47^ used UV-C disinfection at varying doses and showed that even at a moderately high dose (200 mJ/cm^2^), there is a difference of 2 orders of magnitude in the removal of *tet*(A) and *mec*A, with the former being more resilient. They also concluded that damage to ARGs requires UV-C doses at least 1 order of magnitude higher than that required for bacterial inactivation^47^ and thus substantially increases operating costs for UV-C disinfection.

Chlorination disinfection at full scale is also not very effective.^41^ Even at chlorine doses an order of magnitude higher than those used herein, the removal of selected genes was poor and only at very high chlorine concentrations, a substantial reduction in gene copy numbers was observed.^48^ As for HPC, the optimal catalyst load in photocatalysis systems is usually around 1 g/L for ZnO and commonly used in the range of 0.3–2.0 g/L.^49^ Herein, 0.1 g/L of catalyst was used as this is more realistically implementable at full scale.

The low reduction in genes per unit volume of treated wastewater (Figure 1), coupled with the fact that cultivation methods gave bacterial loads of less than 10 CFU/100 mL indicates that while most of the target *E. coli* are no longer viable, they still could have been relatively intact at a molecular level and thus their DNA was not degraded. Such nonviable cells would still be sampled on the membrane and their DNA would be extracted together with viable/culturable cells. While dead–alive bacterial cell discrimination methods exist,^50^ in a real-life tWW irrigation scenario, these would not be removed prior to irrigation and it is possible that DNAs from nonviable cells are incorporated in the soil microbiome and hence were not excluded in this study. The possibility that bacteria, while viable were not cultivable due to the stress of treatment, could not be excluded too. Similarly, this would be identical to real-life conditions and thus no further modifications were performed.

While both treatment intensities used herein were not effective at substantially degrading the evaluated genes (Figure 1), they were suitable for reaching the established target of *E. coli* of <10 CFU/100 mL. Thus, a difference in composition exists between the secondary WW regime (i.e., composed of high gene copy numbers and high *E. coli* loads) and the two treated WW regimes (i.e., composed of high gene copy numbers and low *E. coli* loads). Even for short irrigation campaigns, such as was the case herein, both treatments were not sufficient to avoid increases in potentially deleterious genes, a phenomenon observed with WW irrigation, which resulted in an increase in gene copy number in soil. The treatment of WW (with either HPC or chlorination) did however result in lower increases of soil gene copy numbers for *intI1* relative to the secondary WW. That is, a statistically significant difference in average soil gene copy numbers among irrigation with secondary WW (3.3 × 10^5^ copies/g), chlorinated tWW (6.0 × 10^4^ copies/g; *p* ≤ 0.0001), and HPC tWW (1.6 × 10^5^ copies/g; *p* = 0.0015) was observed for the most abundant gene in water, *intI1*. Chlorinated and HPC-treated WW also resulted in somewhat higher soil values for *bla*_OXA-10_. In FW samples, both *bla*_OXA-10_ and *qnrS* were not present at quantifiable levels.

While the rate constant of hydroxyl radicals (the main expected radical during HPC treatment) with DNA is up to 9 orders of magnitude higher than that with free active chlorine,^51^ applying these two treatments in what could be considered realistic conditions for wastewater reuse in irrigation did not show any clear-cut advantage for using one disinfection method over the other when considering only soil levels of antibiotic resistance-associated genes. While chlorination produced lower *intI1* soil levels relative to HPC (mean = 1.6 × 10^5^ copies/g vs Cl mean = 6.0 × 10^4^ copies/g *p* = 0.0346), the opposite seems the case for *bla*_OXA-10_. It should be noted that while *intI1* is typically associated with anthropogenic activities, it is still common in soil, and its levels could be attributed to either tWW-borne bacteria or soil-borne bacteria. On the contrary, *bla*_OXA-10_ is lacking in soil and only mostly associated with WW; therefore, its increase is to be considered of WW origin. The fact that soil irrigated with WW treated with chlorination resulted in apparently higher levels of *bla*_OXA-10_ relative to that treated by HPC suggests that the latter treatment better targeting the bacterial hosts of this gene in WW, affecting their subsequent recovery/vitality more strongly than chlorination. Di Cesare et al.^31^ observed that while chlorination is effective in inactivating cells, a small population of bacteria can overcome such stress by increasing cell aggregation, which allows for survival of a fraction of them. The cost and complexity of HPC still preclude it from being used as a large-scale environmental water treatment technology,^52,53^ and even taking into account antibiotic resistance as a distinct goal in treatment, HPC, as used herein, does not show substantial benefits over a conventional treatment. Going forward, if HPC is to become useful, co-treatments, such as photocatalytic ozonation, may provide the necessary performance improvements to justify the higher cost.

The huge discrepancy in rate constants between the principal radicals responsible for treatment in HPC and chlorination results in major differences in the half-lives of the radicals themselves.^54^ Mechanistically this, together with the unselective nature of hydroxyl radicals, results in bacterial inactivation by HPC taking place via the oxidation of lipopolysaccharide and other biocomponents of bacterial cell walls, i.e., externally.^55^ Thus, while hydroxyl radicals are reactive toward DNA,^51,56^ unless the cell is lysed, the reaction that takes place during bacterial inactivation is between the external components and the radicals. HPC, to a lesser degree, also proceeds by direct oxidation of the cell walls with photogenerated electron holes on the surface of the photocatalyst. These electron holes are extremely short lived (<50 ns)^55^ and would not result in any reaction once the treatment is stopped. On the other hand, chlorination via hypochlorous acid has multiple bacterial targets both extra- and intracellular.^57^ At the pH of the wastewater used herein (pH 7.4–7.5), HOCl exists together with its dissociated form OCl^–^ and affects bacterial metabolic processes and membrane permeability, fragments and coagulates proteins, and inactivates enzymes and iron–sulfur clusters.^57,58^ Direct damage to DNA in vivo is not clear even though it is known to take place in vitro.^57^ Disinfection by chlorination also has another major distinction from HPC, that is, the residual active chlorine that, among other things, depends on the initial concentration employed and the quantity of organic matter in the water. Residual chlorine concentration was measured after each chlorination test and was found to be always <0.2 mg/L, 1.5 h after the addition of hypochlorous acid. While this could potentially affect the soil bacteria after irrigation has taken place and hence antibiotic resistance genes, we do not expect it to result in major differences relative to HPC irrigation. Residual chlorine does in fact prevent bacterial regrowth in water, but under the storage conditions for treated WW (both by HPC and chlorination), no *E. coli* regrowth was recorded. The residual chlorine levels, i.e., <0.2 mg/L, are also quite low and declining throughout the irrigation week (the residual chlorine concentration decreased from 0.2 to 0.06 mg/L after stored for 3 days at 4 °C). Upon irrigation, chlorinated WW, with any residual chlorine left, would have reacted with organic matter in the soil. While the soil used herein is poor in organic matter (0.12%),^35^ this is still higher than bacterial biomass in the soil, and probabilistically residual chlorine would react mostly with abiotic organic matter, not bacterial biomass, and hence the effect of residual disinfectant on bacteria and genes in soil is expected to be minimal. Circumstantial evidence for this can also be inferred from the 16S rRNA data of the chlorinated and HPC tWW-irrigated soils. The copy numbers of 16S rRNA, as an indicator of total bacteria present, were not statistically different between HPC (with no residual disinfectant) and chlorination (with residual disinfectant). The differences in the resulting gene copy numbers between chlorination and HPC treatments are thus more probably attributed to the differences in mechanism these treatments have and their activity on different bacterial species present in WW. Differential mortality of bacterial species following treatments will in fact affect the persistence and distribution of their harbored ARGs and associated genes in irrigated soils. The current regulations for WW reuse based solely on indicator bacterial loads are not suitable to cover antibiotic resistance gene abetment, at least under the investigated conditions. A purely biomolecular limit as such could be gene copy number per unit volume of specific genes linked to anthropogenic activity,^31^ for example, *intI1*. However, this would also have its limitations since bacterial loads also contribute to the changes in soil quantities of relevant genes.

While chlorination is known to promote the formation of toxic byproducts such as trihalomethanes and other chlorinated byproducts such as haloacetic acids,^22^ these were not so phytotoxic as to result in drastic differences in plant mass. At residual chlorine levels close to the Italian regulatory level of <0.2 mg/L, stunt plant growth has been observed,^59^ and in fact, chlorinated WW-irrigated plants had a significantly smaller aerial height than fresh water-irrigated plants. However, the chlorinated group was not statistically different from the other wastewater groups and hence the contribution from chlorination is probably not major with respect to other phytotoxic compounds present in WW. While deleterious effects on plant growth are known to take place even at this low level (0.2 mg/L), modeling studies with trichloromethane and trichloroethane as model compounds show a low risk of absorption into plant biomass and transfer to humans via the food chain.^60^

In summary, the results show that as far as the differences in the treatment methods are concerned, both HPC and chlorination resulted in statistically higher values of *intI1* and apparent higher levels for *bla*_OXA-10_ compared to the preirrigation levels. Noteworthy, while *intI1* is typically associated with anthropogenic activities, and its levels in the irrigated soil could be attributed to either tWW-borne bacteria or soil-borne bacteria, *bla*_OXA-10_ is lacking in soil and mostly associated with WW; therefore, its increase in the soil after irrigation is to be considered of WW origin. The fact that soil irrigated with WW treated with chlorination resulted in apparently higher levels of *bla*_OXA-10_ relative to that treated by HPC, suggests that the latter treatment was better targeting the bacterial hosts of this gene in WW, affecting their subsequent recovery/vitality more strongly than chlorination. Although this result may not be sufficient to justify the use of HPC, and AOPs in general, with respect to chlorination, other reasons supporting the implementation of AOPs include the higher efficiency in the degradation of organic microcontaminants,^61^ which have a proven exposure pathway from wastewater irrigation to human bloodstream concentrations,^8^ at levels that are bioactive on the development of a model organism (chicken embryo).^9^

## Supporting Information Available

Irrigation log of all pots throughout the irrigation campaign (Table S1) and XRD spectrum of the synthesized 4% cerium-doped zinc oxide (Figure S1) (PDF)

## Abbreviations

WW: wastewater
tWW: treated wastewater
HPC: heterogeneous photocatalysis
ARB: antibiotic-resistant bacteria
ARG(s): antibiotic resistance gene(s)
AR: antibiotic resistance
UWTP: urban wastewater treatment plant
NTU: nephelometric turbidity unit
COD: chemical oxygen demand
BOD: biological oxygen demand (BOD_5_)
DOC: dissolved organic carbon
C.I.: confidence interval
