# Supplementary material for: Changes in Antibiotic Resistance Gene Levels in Soil after Irrigation with Treated Wastewater: A Comparison between Heterogeneous Photocatalysis and Chlorination
Source: Environ Sci Technol. 2020 May 15;54(12):7677–86. doi: 10.1021/acs.est.0c01565 (PMC8007107; doi:10.1021/acs.est.0c01565)
Supplement: Supplementary file 1 — es0c01565_si_001.pdf [file es0c01565_si_001.pdf]

Supporting Information

Changes in antibiotic resistance gene levels in soil after irrigation with treated wastewater: a comparison between heterogeneous photocatalysis and chlorination

Ian Zammit†, Roberto B. M. Marano ‡§ , Vincenzo Vaiano‖, Eddie Cytryn ‡, Luigi Rizzo†**

† Department of Civil Engineering, University of Salerno, Via Giovanni Paolo II 132, 84084 Fisciano, Italy;

‡Department of Soil Chemistry, Plant Nutrition and Microbiology, Institute of Soil, Water and Environmental Sciences, Volcani Center, Agricultural Research Organization, Rishon LeZion, Israel.

§ Department of Agroecology and Plant Health, The Robert H. Smith Faculty of Agriculture, Food and Environment, The Hebrew University of Jerusalem, Rehovot, Israel.

ǁ Department of Industrial Engineering, University of Salerno, Via Giovanni Paolo II 132, 84084 Fisciano, Italy

* corresponding author l.rizzo@unisa.it; eddie@volcani.agri.gov.il

The Supporting Information is 3 pages long with 1 table and 1 figure

Date	Irrigated Series				mg of nitrogen added	Fertilised Series	Days since Start
	FW (mL)	WW (mL)	OCl (mL)	HPC (mL)			
2018-10-18	800	800	800	800			0
2018-10-21	100	100	100	100			3
2018-10-23	150	150	150	150			5
2018-10-25	250	250	250	250			7
2018-10-28	150	150	150	150			10
2018-10-29	150	150	150	150	8.25	All	11
2018-10-31	150	150	150	150			13
2018-11-01	150	150	150	150			14
2018-11-04	200	200	200	200			17
**2018-11-04**	**Soil sampling of all pots (pre-tWW)**						
2018-11-05			180				18
2018-11-06	180	180		180			19
2018-11-08	400	400	400	400			21
2018-11-11		170		170	9.35	HPC, WW, FW	24
2018-11-12	170		170		9.35	OCl	25
2018-11-14	170	170	170	170			27
2018-11-15	300	300	300	300			28
2018-11-18	175	175	175				31
2018-11-19				175			32
2018-11-21	200	200	200	200			34
2018-11-22	250	250	250	250			35
2018-11-25	170	170	170				38
2018-11-26	100	100	100	300	5.5	All	39
2018-11-29	420	420	420	390			42
2018-12-02	185	185		185	10.175	HPC, WW, FW	45
2018-12-03			185		10.175	OCl	46
2018-12-05	250	250	250	250			48
2018-12-09	350	350	350				52
2018-12-10				400			53
2018-12-11	300	300	300	250			54
2018-12-12	110	110	110	110			55
**2018-12-13**	**Soil sampling of all pots (post-tWW)**						
Total	5830 mL	5830 mL	5830 mL	5830 mL	27.8 mg		

Table S1 – irrigation log of all pots

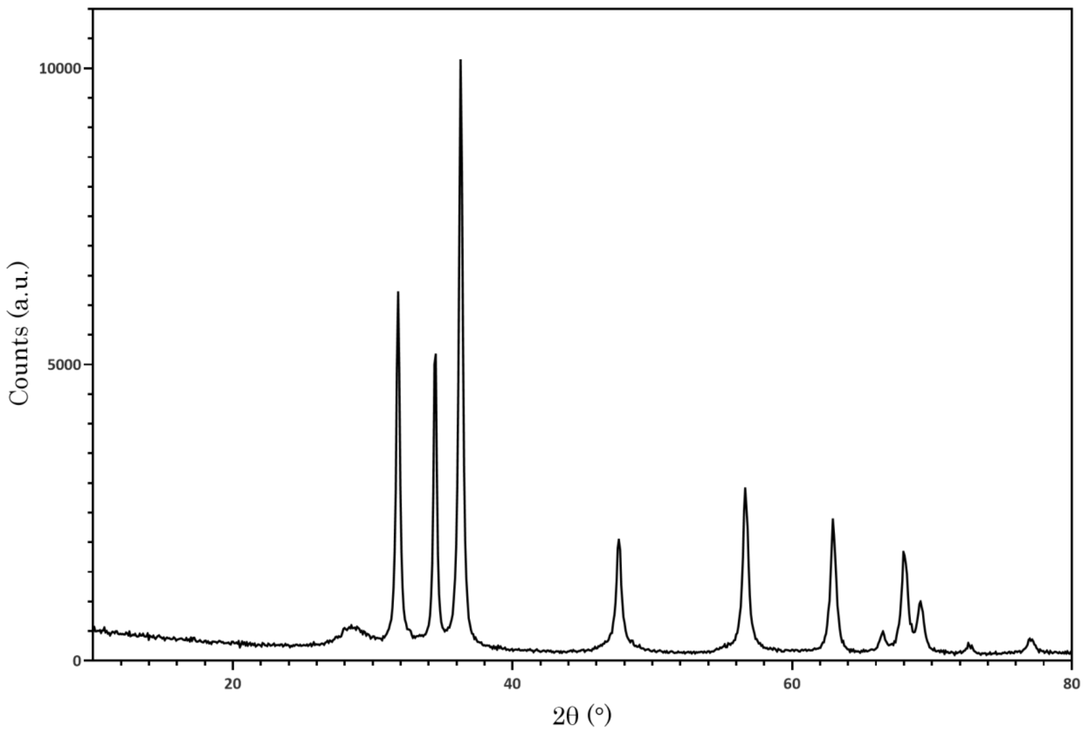

Figure S1 – XRD of the synthesised photocatalyst - 4% cerium doped zinc oxide
